# Bilateral Pleural Effusion in Rheumatoid Arthritis: Think Beyond the Obvious

**DOI:** 10.31138/mjr.33.3.369

**Published:** 2022-09-30

**Authors:** Ilias E. Dimeas, Sotirios I. Sinis, George E. Dimeas, Zoe Daniil

**Affiliations:** Department of Respiratory Medicine, Faculty of Medicine, University of Thessaly, Biopolis, Larissa, Greece

**Keywords:** rheumatoid arthritis, bilateral pleural effusions, tuberculous pleurisy

**Figure 1.**
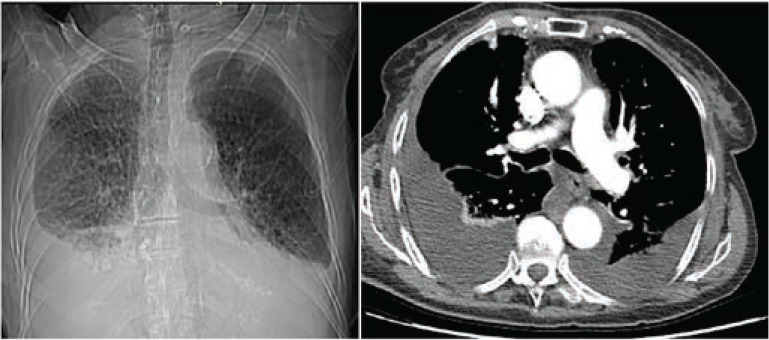
Computed tomography pulmonary angiogram.

**Figure 2.**
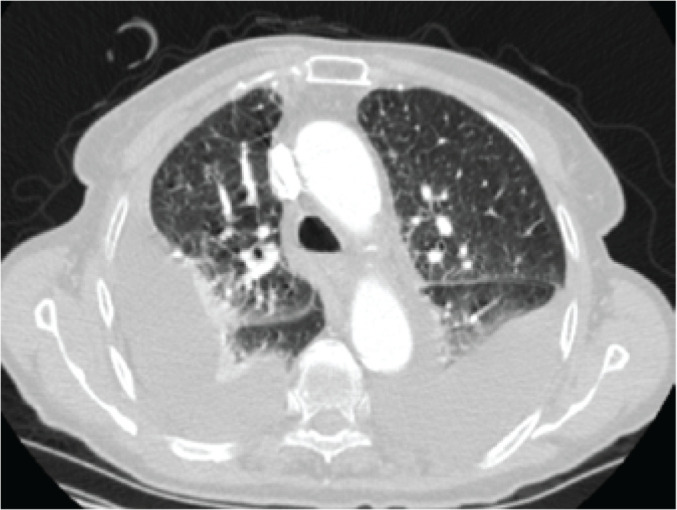
Full body computed tomography.

**Figure 3.**
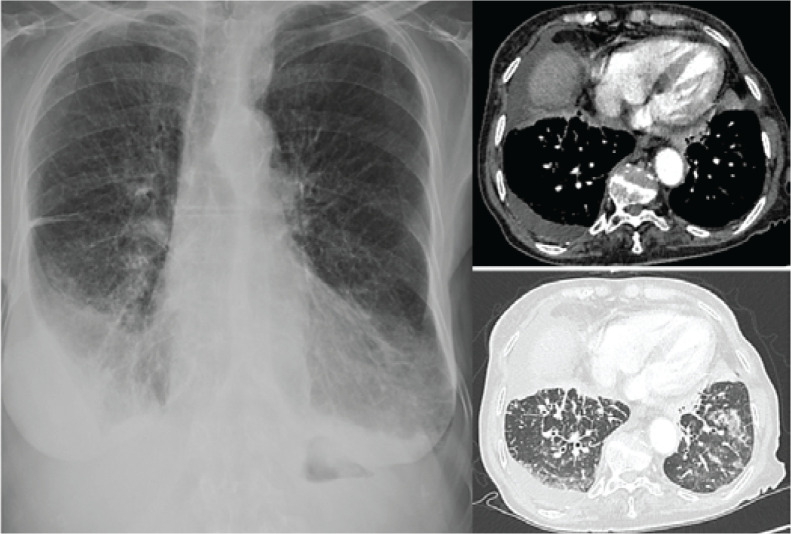
Patient follow-up.

An 86-year-old woman, non-smoker, with a medical history of rheumatoid arthritis for 20 years (stable at 5mg of prednisolone) was referred from a peripheral hospital to the Department of Respiratory Medicine of our tertiary hospital for further evaluation of bilateral pleural effusions with the provisional diagnosis of rheumatoid arthritis pleurisy. The patient reported frailty starting two months prior, low-grade fever, and mild small joints’ arthralgias for one week and pleurodynia in her right hemithorax starting two days ago. A computed tomography pulmonary angiogram (**Figure 1**) ruled out pulmonary embolism revealing bilateral pleural effusions and a diagnostic thoracocentesis showed a predominantly neutrophilic exudate with normal pH and quite low glucose level (71mg/dl). A high titre of rheumatoid factor in the neutrophilic exudate plus the medical history of rheumatoid arthritis set the provisional diagnosis of a rheumatoid arthritis’ pleurisy and the patient was referred. Upon our clinical examination, diminished lung sounds in both lung bases were found without additional lung sounds. A new diagnostic thoracocentesis was performed which revealed surprisingly a predominantly lymphocytic exudate with normal pH but again quite low glucose level (62mg/dl). The adenosine deaminase (ADA) value was high (84 IU/L) highly suggestive of tuberculosis, mesothelioma, or lymphoma. A full body computed tomography (**Figure 2**) was performed which was negative for solid tumour, pleural thickening or enlarged lymph nodes. The Ziehl-Neelsen staining of the pleural fluid revealed an acid-fast organism and the next day the polymerase chain reaction was positive for mycobacterium tuberculosis. A three regimen antituberculosis therapy was initiated and in the follow up of the patient (**Figure 3**) bilateral pleural effusions were reduced. Pleural exudate in patients with known rheumatoid arthritis should not be always attributed to their condition, as thorough investigation might reveal tuberculosis or malignancy.

